# Lipidomic profiling unveils sex differences in diabetes risk: Implications for precision medicine

**DOI:** 10.1111/eci.70137

**Published:** 2025-10-24

**Authors:** Ana F. Pina, Maria João Meneses, Fabrizia Carli, Rogério T. Ribeiro, Luís Gardete‐Correia, José M. Boavida, João F. Raposo, Amalia Gastaldelli, M. Paula Macedo

**Affiliations:** ^1^ iNOVA4Health, NOVA Medical School, Faculdade de Ciências Médicas, NMS, FCM Universidade NOVA de Lisboa Lisbon Portugal; ^2^ ProRegeM PhD Program, NOVA Medical School, NMS Universidade Nova de Lisboa Lisbon Portugal; ^3^ APDP – Diabetes Portugal Education and Research Center Lisbon Portugal; ^4^ National Research Council (CNR) Institute of Clinical Physiology (IFC) Pisa Italy; ^5^ Portuguese Society of Diabetology Lisbon Portugal; ^6^ Scuola Superiore Sant'Anna Pisa Italy

**Keywords:** clustering, diabetes, dysmetabolism, heterogeneity, lipidomics

## Abstract

**Aims/Hypothesis:**

Type 2 diabetes is a multifactorial condition whose greatest impact comes from its complications. We hypothesized that distinct insulin‐derived mechanisms and lipid profiles discriminate sex differences and can be used to identify subjects at higher risk to develop diabetes‐related complications.

**Methods:**

The PREVADIAB2 study evaluated metabolic alterations after 5 years in individuals initially free of Type 2 Diabetes (PREVADIAB1). In this analysis, 953 participants were stratified into clusters using hierarchical clustering based on insulinogenic index (IGI), fasting insulin secretion rate, HOMA‐IR, and fasting insulin clearance. A subset of participants (*n* = 488) had their lipidome assessed using LC/MS‐QTOF.

**Results:**

Four clusters were identified: Liver Sensitive (LS), Pancreas Glucose Sensitive (PGS), Insulin Deficient (ID), and Insulin Resistant (IR), each with distinct dysglycemia risk. While metabolic features were similar across sexes, the parameter thresholds differed, resulting in sex‐specific lipidomic profiles. Women exhibited higher levels of circulating dihydroceramides (5.3 ± 1.9 vs. 4.7 ± 1.8, *p* < .001), associated with de novo ceramide synthesis, and elevated sphingomyelins (SM), suggesting altered lipid metabolism. Conversely, the ceramide‐to‐SM ratio was higher in men (1.04 ± .21 vs. .90 ± .18, *p* < .001). Except for the LS cluster, all other clusters exhibit distinct lipid signatures associated with metabolic dysfunction, further accentuated by specific lipid profile sex differences.

**Conclusions:**

Distinct insulin‐related metabolic features and sex identify different phenotypes with distinct lipidome profiles, highlighting the need to place prediabetes in a broader context of metabolism beyond glucose.

## Graphical abstract

This study uncovered sex‐specific metabolic and lipidomic signatures of prediabetes. In PREVADIAB2 (*n* = 953), clustering by insulin traits revealed four phenotypes. Lipidomics showed women enriched in dihydroceramides and sphingomyelins, while men displayed a higher ceramide/sphingomyelin ratio. These sex‐metabolic phenotypes expose distinct lipidomes, underscoring the need to view prediabetes as a metabolic disorder beyond glucose.

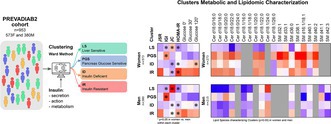

## 1 INTRODUCTION

The effective management of type 2 diabetes requires a comprehensive approach. Besides hyperglycemia, there are other risk factors associated with diabetes‐related complications, for example, dyslipidemia and hypertension.^1^ Despite the current diagnostic criteria for type 2 diabetes primarily focusing on glycemia, its development is attributed to abnormalities in insulin secretion, action or metabolism that may be altered even when still in normoglycemia.^2^, ^3^ Furthermore, insulin plays a role in lipid metabolism, which is intricately connected to glucose metabolism.^4^

The complexity of diabetes and its subphenotypes^5^, ^6^ requires an approach that considers insulin mechanisms, metabolites like glucose and lipids, and their impact on complications.^7^

Although in recent years, lipidomics has emerged as a valuable tool for studying dysmetabolism, enabling the identification of lipid species that are altered in diabetes and comorbidities,^8^, ^9^ only a few studies have stratified individuals with or at risk of type 2 diabetes using lipidomics.^10^, ^11^

Indeed, lipids, including diacylglycerols (DAGs), triglycerides (TGs), ceramides (CERs), sphingomyelins (SMs) and phosphatidylcholines (PC), can serve as disease biomarkers.^12^ From these, studies have pinpointed ceramides as harmful to tissues and linked to conditions like metabolic dysfunction‐associated steatotic liver disease (MASLD). Notably, dihydroceramides (DhCer), which are precursors to CERs, were found to be elevated in plasma through population‐based lipidomic analysis. This finding indicates their potential to predict diabetes up to 9 years before its onset.^13^ Importantly, lipidomic profiles differ by sex and age in healthy individuals, suggesting distinctions in pre/diabetes or its progression and contributing to sex‐specific risks for complications.^14^

This study, PREVADIAB2, is a follow‐up to PREVADIAB1 and was conducted with subjects who had no diabetes in PREVADIAB1. Using clustering analysis, we aimed to define phenotypes with distinct glycemic patterns and lipidomic profiles based on diabetes‐related pathophysiological mechanisms within the PREVADIAB2 population. We further hypothesized that sex‐specific differences, particularly in lipid profiles, could aid in distinguishing subgroups and explain unique physiological features. Our goal was to determine whether early changes in lipid and glycemic profiles are linked to diabetes pathophysiology and related alterations in insulin secretion, action and metabolism.

## 2 MATERIALS AND METHODS

### 2.1 Population

The study population consists of 953 participants from PREVADIAB2, which is a follow‐up of PREVADIAB1.^6^, ^15^ Five years after the initial study, 1088 subjects without diabetes in PREVADIAB1 were randomly selected to participate in PREVADIAB2. All participants gave their voluntary consent and signed written informed consent forms. Ethical permits were obtained from the *Associação Protectora dos Diabéticos de Portugal* Ethics Committee. The study followed the Declaration of Helsinki and was approved by the *Autoridade Nacional de Protecção de Dados* (3228/2013).

Only individuals with complete data for the variables used in the cluster analysis described below were included in the study. Additionally, individuals who were on antidiabetic medication were excluded (Figure S1). For the retrospective analysis of glycemia progression, participants with missing data were not included.

### 2.2 Clinical and Biochemical Parameters

Evaluation of the PREVADIAB2 participants was previously described.^6^ Briefly, subjects underwent a 75 g oral glucose tolerance test (OGTT), and glucose, insulin, C‐peptide, and free fatty acids blood levels were measured at 0, 30 and 120 min of the OGTT (Detailed methodology in Appendix S1). The (pre)diabetes status was determined using the 2019 WHO criteria (https://www.who.int/publications/i/item/classification‐of‐diabetes‐mellitus accessed April 25th, 2024).

### 2.3 Biochemical and Metabolic Indexes

Fasting insulin secretion rate (_f_ISR)^16^ and insulinogenic index (IGI)^17^ were used to evaluate beta‐cell insulin response. Insulin clearance (L.min^−1^) was calculated as the ratio of peripheral insulin disposal rates (tissue extraction and degradation) and circulating insulin concentrations.^16^ The suppression of insulin clearance during OGTT^16^ was presented as the slope of insulin clearance from 0 to 30 min (∆(0–30)IC), and additionally, by the value of insulin clearance at 120 min (IC 120 min). HOMA‐IR2 was used to evaluate insulin resistance at fasting. Organ‐specific insulin resistance indexes were also calculated.^18^ hepatic‐IR [AUC(0–30)Insulin × AUC(0–30)Glucose] and adipo‐IR [FFA_0min_ × Insulin_0min_]. To evaluate pancreatic function, we considered the OGTT‐Disposition Index (DI).^16^ NAFLD‐FLS, a surrogate index of MASLD, was calculated as previously described.^19^

### 2.4 Lipidomic analysis

Serum lipidome was analysed by high resolution mass spectrometry (UHPLC‐QTOF; Agilent Technologies). The analysis included triglycerides, CERs, SM, PC and lysophosphatidylcholines (LPC) quantified with an internal standards mixture containing N‐heptadecanoyl‐D‐erythro‐sphingosylphosphorylcholine (SM(d18:1/17:0)), N‐heptadecanoyl‐D‐erythro‐sphingosine (Cer(d18:1/17:0)), 1,2‐diheptadecanoyl‐sn‐glycero‐3‐phosphocholine (PC(17:0/17:0)) and 1‐heptadecanoyl‐2‐hydroxy‐sn‐glycero‐3‐phosphocholine (LPC(17:0); Avanti Polar Lipids) and tripentadecanoin (TG(15:0/15:0/15:0); Larodan AB).

10 μL of serum were mixed with 10 μL of an internal standards mixture and 150 μL of cold methanol to precipitate proteins. Then, sample was centrifuged at 14000 rpm at 4°C for 20 minutes and 1 μL of the supernatant was injected in UHPLC‐QTOF system, combining a 1290 Infinity LC system and a 6545 quadrupole time‐of‐flight mass spectrometer (QTOF), interfaced with a dual jet stream electrospray (dual ESI) ion source in positive mode. Lipids were separated by ZORBAX Eclipse Plus C18 2.1 × 100 mm 1.8 μm column (Agilent Technologies). The solvent system included A: ultrapure water with .1% formic acid and B: LC/MS grade isopropanol:acetonitrile (1:1, v:v). The gradient ramp for mobile phase B was: 35% at start, then 80% from 0 to 2 min, 100% from 2 to 9 min, and maintained at 100% from 9 to 16 min, with a postrun of 6 min to return to start conditions. The flow rate was .4 mL/min from 0 to 9 min and .6 mL/min from 9 to 16 min. The NIST SRM 1950, a frozen human plasma sample, was used as a reference material for lipidomics measurements and as quality control was used an aliquot of a pool obtained from the sample set.

The data processing included peak detection, integration, peak alignment, normalization, and identification using Profinder MassHunter B.08.00 (Agilent Technologies) software. Lipids were identified using an internal customized spectral library. The data were normalized using the internal standards representative of each class of lipid present in the samples: the intensity of each identified lipid was normalized by dividing it by the intensity of its corresponding standard and multiplying it by the concentration of the standard. Only lipids that were above the limit of quantification in more than 60% of the samples were reported for this data set.

### 2.5 Cluster and Statistical Analyses

During the preprocessing stage, severe outliers were assessed using a multidimensional approach, and the data was centered and scaled. Afterward, the cluster analysis was done with the agglomerative hierarchical clustering algorithm with the Ward method (stats::hclust in R), with the following variables as input: _f_ISR and IGI to estimate insulin secretion during fasting and the first phase of the OGTT, respectively; _f_IC to measure fasting insulin clearance; and HOMA‐IR as an indicator of overall insulin resistance. The optimal number of clusters was determined (NbClust, R), employing the majority rule. Cluster stability was assessed by calculating the mean Jaccard coefficient. A sensitivity analysis was conducted separately for normoglycemia and dysglycemia to assess if the clustering patterns remained stable across these subgroups.

Lipidomic data analysis was performed (lipidr, R^20^) after being normalized and log2‐transformed. Outliers were assessed and filtered using a multidimensional evaluation (Figure S2). PC C36:6 was excluded from the analyses as it had several outliers and few other outlier samples identified with PCA (Figure S2). After applying a stratified sampling procedure across clusters and gender strata to ensure balanced representation of subgroups, and following data preprocessing, 103 lipid species in 273 women and 215 men were analysed. Lipidomic‐clusters association was assessed adjusting for age, BMI, and glycemia subgroups. Benjamini‐Hochberg (BH) was used to adjust for multiple comparison.

Except for the lipidomic analysis, comparison between group means (clusters and sex) was performed with Kruskal–Wallis and Wilcoxon‐Mann–Whitney tests for continuous variables; Chi‐square test for the comparison of categorical variables. Bonferroni correction was used for multicomparison, when applicable.

## 3 RESULTS

### 3.1 Population metabolic characterization and overall sex differences

We analysed 953 subjects from the PREVADIAB2 cohort that had no diabetes in PREVADIAB1 (5 years prior), with a mean age of 61 ± 13 years; 60% were women (Table 1).

**TABLE 1:** Summary statistics of the population.

	All	Women	Men	*p*‐Value
Total *n* (%)	953	573 (60)	380 (40)	‐
Normoglycemia *n* (%) Prediabetes *n* (%) Diabetes *n* (%)	697 (73) 208 (22) 48 (5)	421 (73) 125 (22) 27 (5)	276 (73) 83 (22) 21 (5)	.85
Age, Years	61 ± 13	61 ± 13	61 ± 13	.87
BMI, kg/m^2^	27.3 ± 4.3	27.7 ± 4.6	26.9 ± 3.6	.01
Fast‐ISR, pmol/min	**170 ± 78**	**164 ± 72**	**179 ± 84**	.**007**
IGI	.**90 ± .9**	.**94 ± .9**	.**86 ± .9**	.**005**
Fast‐IC, L/min	**4.2 ± 1.5**	**4.0 ± 1.4**	**4.6 ± 1.7**	**<.001**
HOMA‐IR	**1.8 ± 1.2**	**1.8 ± 1.2**	**1.8 ± 1.2**	.**75**
ISI_0‐120_, Log10	67 ± 19	66 ± 18	69 ± 20	.05
Adipo‐IR	27 ± 19	27 ± 19	26 ± 18	.5
Hepatic‐IR, ×10^4^	7.3 ± 5.4	7.3 ± 5.1	7.2 ± 5.9	.05
Disposition Index, mmol^−1^	2.4 ± 2.5	2.4 ± 2.6	2.2 ± 2.3	.12
NAFLD‐FLS	−1.2 ± 1.2	−1.3 ± 1.2	−1.2 ± 1.3	.51
Glucose 0 min, mmol/L	5.1 ± .7	5.1 ± .6	5.3 ± .8	<.001
Glucose 30 min, mmol/L	8.6 ± 1.8	8.5 ± 1.7	8.8 ± 1.9	.04
Glucose 120 min, mmol/L	6.5 ± 2.2	6.6 ± 2.1	6.3 ± 2.4	<.001
Total cholesterol, mg/dL	201.0 ± 37.1	204.0 ± 35.5	197.0 ± 39.1	.009
LDL‐c, mg/dL	138.0 ± 30.5	138.0 ± 29.5	137.0 ± 32.1	.48
HDL‐c, mg/dL	53.3 ± 12.3	56.0 ± 12.1	49.4 ± 11.4	<.001
Triglycerides, mg/dL	117.0 ± 60.3	112.0 ± 53.7	124.0 ± 68.5	.03

*Note*: Values are reported as mean ± SD for continuous parameters and as count and percentages for categorical parameters. Comparison between sex was performed with the Mann–Whitney test for continuous variables and with the chi‐square test for categorical variables. In bold are parameters informing cluster analysis.

Women and men exhibit differences in parameters used to inform and to profile the clusters. When compared to men, women had higher BMI and higher IGI despite lower insulin secretion at fasting. Additionally, women tended to have higher hepatic‐IR than men and lower ISI_0‐120_. Consistently, women had lower IC than men. Finally, women had lower glycemic values at fasting and at 30 min but higher levels at 120 min of the OGTT (Table 1).

### 3.2 Clusters metabolic characterization

We sought to validate if men and women exhibit the same cluster centroids related to insulin mechanisms (_f_ISR, IGI, HOMA‐IR and _f_IC) and potential differences in their lipidomic profiles (Table S1).

The population was stratified based on three mechanisms related to diabetes pathophysiology: insulin secretion (_f_ISR and IGI to estimate insulin secretion at fast and during the first phase of the OGTT), resistance (HOMA‐IR), and fasting insulin clearance (_f_IC) that reflects mainly hepatic IC (Figure S1). We initially examined differences among clusters in the entire population and then separately for women and men.

In overall population, the best number of clusters was four, named according to their metabolic profiles (Table S2). The overall pattern of cluster characteristics was preserved within subgroups. Nonetheless, there were few differences in the centroids of the clusters when the subgroups were compared, namely regarding IGI (Table S2). The liver‐sensitive (LS) cluster demonstrated the highest IC and the lowest hepatic‐IR. The pancreas glucose sensitive (PGS) cluster exhibited the highest DI and IGI. Nevertheless, the PGS cluster had higher HOMA‐IR and suppressed IC compared with the LS cluster. The insulin deficient (ID) cluster displayed lower _f_ISR and IGI, but had higher HOMA‐IR than the LS cluster. The insulin resistant (IR) cluster showed suppressed IC, the highest HOMA‐IR, and consistently the highest _f_ISR, though not IGI.

When analysing data by sex, we found similar patterns in clusters for both men and women compared to the overall population, but their cluster centroids differed (Table S2). Therefore, separate cluster analyses were conducted for each sex due to potential differences in parameter cut‐off levels. Variable importance rankings differed between the overall population and each sex, revealing distinct metabolic patterns (Table S1). Notably, HOMA‐IR was highly important in women but least important in men.

**FIGURE 1:**
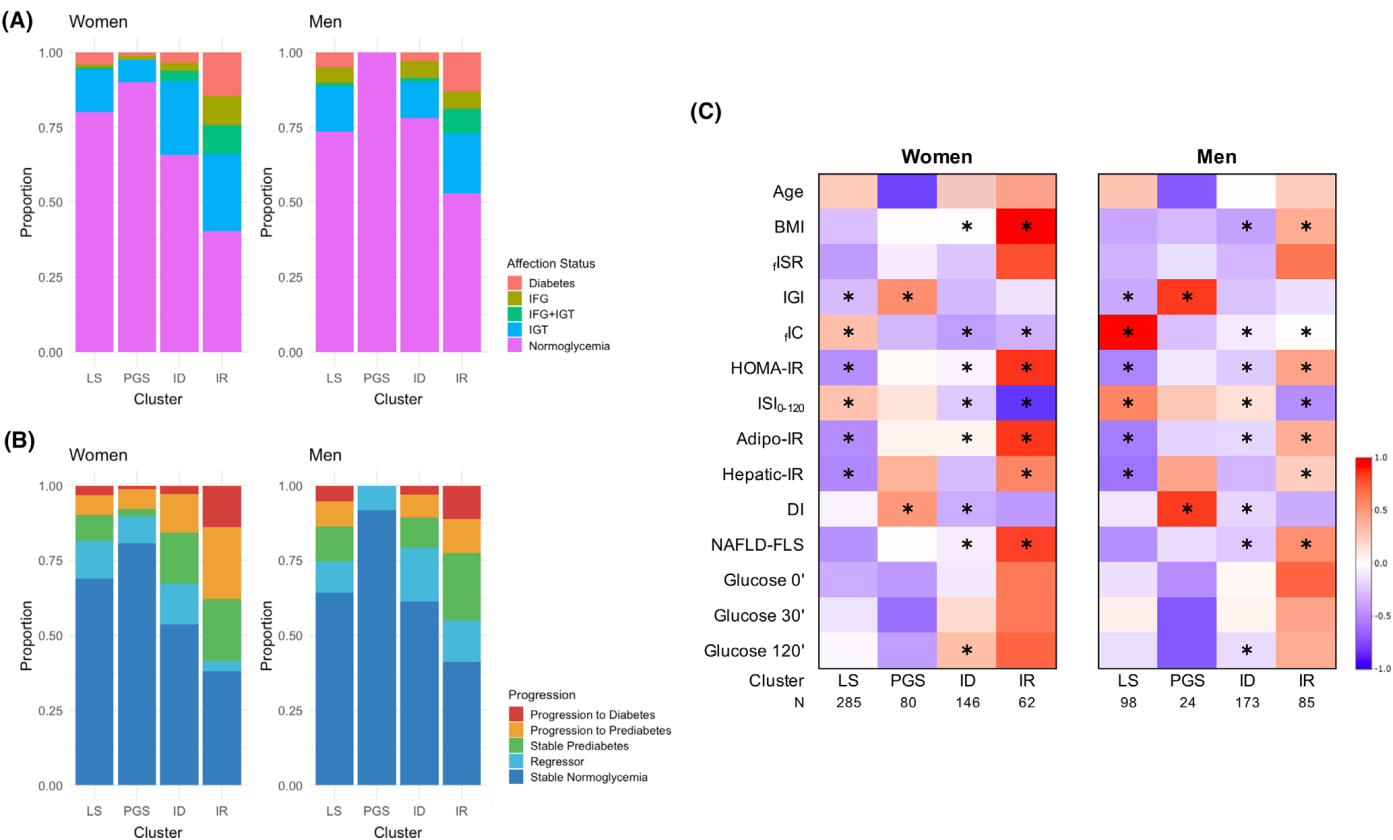
(A) Proportion of glycemia classes in the clusters by sex. (B) Retrospective evaluation of glycemia evolution within the clusters, by sex. Proportion of individuals that have progressed (from normoglycemia to prediabetes or type 2 diabetes, or from prediabetes to type 2 diabetes), regressed (from prediabetes to normoglycemia), or maintained their glycemic status in a 5‐year period (from PREVADIAB1 study to PREVADIAB2). (C) Cluster's profile by sex. Heatmap representing the scaled mean of each parameter for the entire cohort. * represents the significant differences (*p* < .05) in women vs. men within each cluster and for each parameter; detailed information on Table S2. N for each cluster is depicted in panel C. Cluster names: Liver‐sensitive (LS); pancreas glucose sensitive (PGS); insulin deficient (ID); insulin resistant (IR). BMI, body mass index; fISR, fasting insulin secretion rate; IGI, insulinogenic index; fIC, fasting insulin clearance; HOMA‐IR, Homeostatic Model Assessment for Insulin Resistance; ISI, insulin sensitivity index; Adipo‐IR, adipose tissue insulin resistance index; Hepatic‐IR, hepatic insulin resistance index; DI, disposition index; NAFLD‐FLS, non‐alcoholic fatty liver disease‐fatty liver score.

Clusters showed distinct risks for hyperglycemia in both sexes, with the PGS cluster showing the lowest risk of dysglycemia and the IR cluster the highest risk (Figure 1A and Table S3). A 5‐year retrospective analysis of glycemic class evolution was performed (Figure 1B), considering that all individuals had no diabetes 5 years prior to this study. More individuals progressed to prediabetes or diabetes in the IR cluster, especially among women compared to men. ID women also showed a high number of progressors, while the PGS cluster had the highest proportion of individuals remaining normoglycemic. Overall, the incidence of prediabetes and diabetes was higher in women than in men.

The heatmap displaying cluster profiling based on metabolic characteristics separately for women and men (Figure 1C) shows that men from the LS cluster had higher IC and _f_ISR and lower IGI and insulin‐resistance indexes (overall and organ‐specific) than women (Table S2). However, men in the PGS cluster had a higher insulinogenic index and disposition index. Men from the ID cluster had lower _f_ISR than women, along with lower IR indexes and higher IC and DI. Additionally, ID men had lower BMI than ID women. Finally, compared to women from the IR cluster, men also had lower BMI, insulin resistance indexes and higher IC. However, they did not differ in insulin secretion indexes–_f_ISR and IGI (Table S2).

### 3.3 Population lipidomic characterization and overall sex differences

From the 103 evaluated species, women and men showed distinct lipidomic profiles. In women, SMs were higher than in men. The CER to SM ratio, which might be associated with insulin secretion defects,^21^ was higher in men (.90 ± .18 vs. 1.04 ± .21 for women and men, respectively; *p* < .001). Additionally, LPCs and the LPC/PC ratio, associated with an increased risk of an inflammatory profile,^22^ were higher in men than in women (4.12 ± 4.6 vs. 1.4 ± .6, *p* < .001). Women had higher levels of long‐chain (C14:0–C20:0) and very‐long‐chain dihydroceramides (C22:0–C25:0) than men, which are related to the de novo ceramide synthesis (5.3 ± 1.9 μmol/L vs. 4.7 ± 1.8 μmol/L, *p* < .001). In contrast, men had higher very and ultra‐long‐chain ceramides (10.2 ± 3.4 μmol/L vs. 11.6 ± 4.0 μmol/L, *p* < .001).

### 3.4 Clusters lipidomic characterization

**FIGURE 2:**
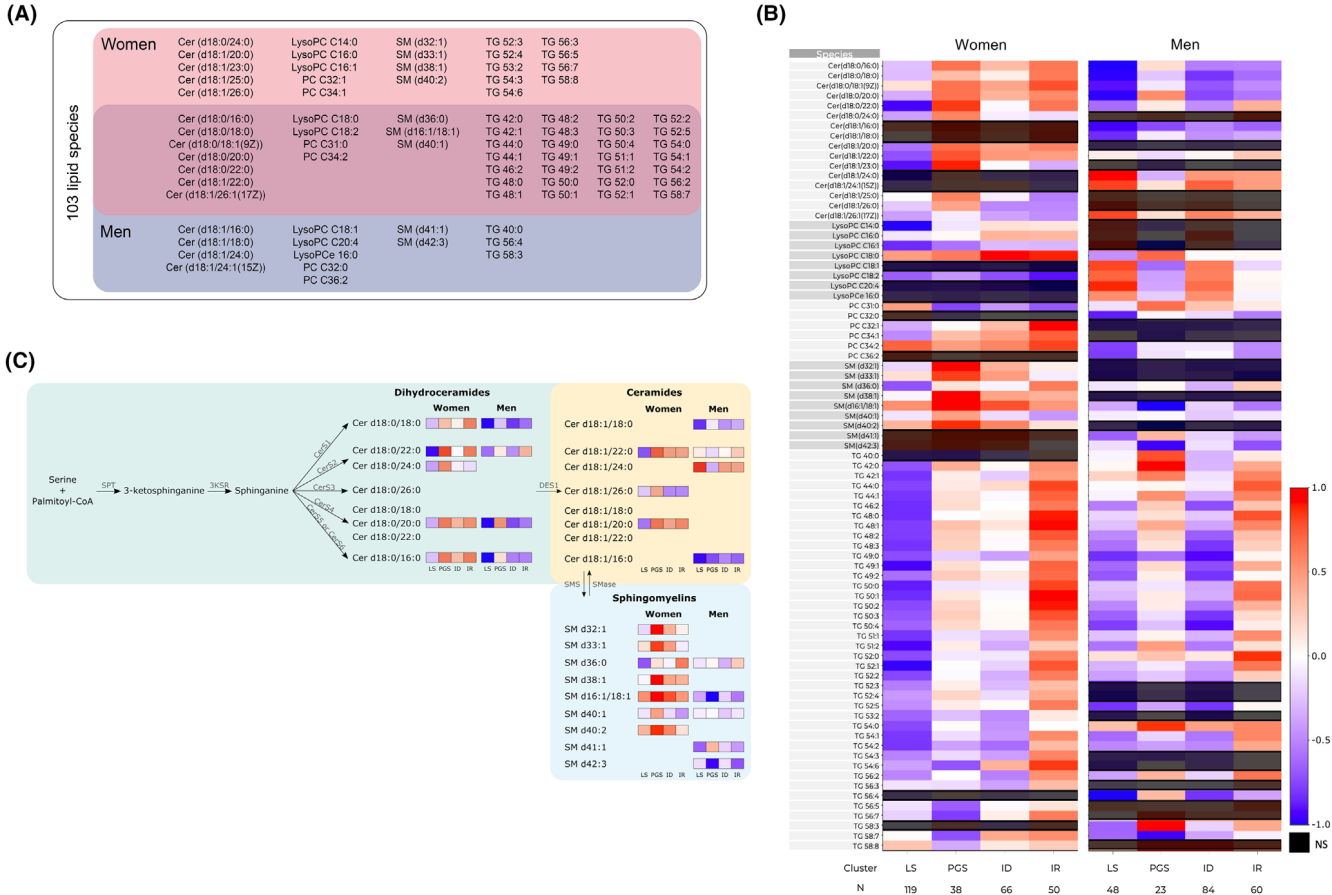
Lipidomics cluster's profiling. (A) Lipid species associated with clusters. The analysis was done individually for each sex. From the 103 lipid species, 23 were associated with female clusters, 14 with male clusters, and 42 associated with clusters of both sexes. (B) Heatmap representing the scaled mean of each lipid specie in the overall population. (C) Graphical representation of ceramide synthesis via de novo synthesis and sphingomyelin hydrolysis. N for each cluster is depicted in panel B. Lipid Species: CER, ceramides; LysoPC, lysophosphatidylcholine; PC, phosphatidylcholine; SM, sphingomyelin; TG, triglycerides. Clusters Names: ID, insulin deficient; IR, insulin resistant; LS, liver sensitive; PGS, pancreas glucose sensitive. 3KSR, 3 ketosphinganine reductase; CERS, Ceramide Synthase; DES1, Dihydroceramide desaturase; NS, non‐significant; SMase, Sphingomyelinase; SMS, Sphingomyelin synthase; SPT, Serine Palmitoyltransferase.

Distinct lipidomic profiles were observed throughout the clusters and between sexes (Figure 2, Figures S3 and S4). From the evaluated lipid species, 42 were associated with both sexes; 23 were associated only with women clusters, and 14 lipids were only associated with men clusters (Figure 2A; Table S4).

Figure 2B represents the clusters profiling for the lipid species that were found to be significantly associated with clusters in women and/or in men (*p* < .05, BH correction for multiple comparisons) in the overall population (for glycemia subgroups separately see Figure S5).

#### 3.4.1 Liver Sensitive Cluster (LS) lipidomic signature

Women and men, in comparison to other clusters, presented lower overall serum concentrations of DhCer; specifically, Cer(d18:0/16:0), Cer(d18:0/18:0), Cer(d18:0/20:0), Cer(d18:0/22:0), suggesting a decrease of de novo hepatic ceramide synthesis, which has been associated with reduced steatosis. Moreover, LS presented low levels of SM36:0, particularly in women, which has been shown to be decreased in individuals with less severe stages of liver disease.^23^ Indeed, this cluster showed low Hepatic‐IR and NAFLD‐FLS score. Furthermore, women exhibited remarkably low levels of triglycerides (TG), specifically TG48:0 and TG48:2, suggesting a potential decrease in hepatic de novo lipogenesis (DNL).^24^ Similarly, men had low TG (e.g., TG48:2 and TG56:4) compared to the remaining clusters. Interestingly, men in this cluster revealed high levels of CERs (Cer(d18:1/24:0), Cer(d18:1/24:1), Cer(d18:1/26:1)) and high LPC18:2 despite low LPC18:0.

#### 3.4.2 Pancreas Glucose Sensitive Cluster (PGS) lipidomic signature

Even though this cluster revealed the lowest glucose levels, lipid homeostasis was disturbed and associated with low insulin clearance and high hepatic insulin resistance. Subjects within the PGS cluster exhibited elevated levels of DhCer and CER, particularly among women. Notably, this cluster displayed the highest levels of DhCer in women, specifically Cer(d18:0/16:0), Cer(d18:0/18:0), Cer(d18:0/20:0) and Cer(d18:0/22:0), suggesting increased de novo Cer synthesis. Accordingly, CER levels were significantly elevated, including Cer(d18:1/20:0), Cer(d18:1/22:0), Cer(d18:1/23:0), Cer(d18:1/25:0), and Cer(d18:1/26:0). This group also presented high levels of SM, particularly in women (SM(d32:1), SM(d33:1), SM(d38:1), SM(d16:1/18:1), SM(d40:1), SM(d40:2)). Finally, the PGS cluster had low LPC14:0, LPC16:1 and LPC18:2. Nevertheless, LPC16:0 and LPC18:0 were higher, which notably have been associated with decreased type 2 diabetes risk.^25^ Furthermore, it is noteworthy that TG levels, particularly TG48:2, having the highest value in men, were increased, implying a rise in DNL.

#### 3.4.3 Insulin Deficient Cluster (ID) lipidomic signature

The lipidomic profile within the ID cluster resembled that of the LS cluster in men showing low levels of DhCer and CERs, except for Cer(d18:1/22:0), but not in women that show increased DhCer (d18:0/20:0 and d18:0/16:0) and Cer(d18:1/22:0). Women also displayed higher Adipo.IR. Additionally, the ID cluster displayed reduced hepatic insulin resistance (Hepatic‐IR) and metabolic‐associated steatotic liver disease (NAFLD‐FLS).

Notably, despite the low levels of certain triglycerides (TG), such as TG49:0 and TG50:4, observed in men, women displayed a nuanced response. Some TGs exhibited a slight increase, while others, like TG56:2, TG56:3 and TG56:4, were found at their lowest levels. In comparison, men exhibited higher insulin sensitivity and improved insulin clearance (Table S2).

On the other hand, women in the ID cluster had elevated levels of LPC C18:0 compared to other clusters, indicating a unique lipidomic profile in females.

#### 3.4.4 Insulin Resistant Cluster (IR) lipidomic signature

**FIGURE 3:**
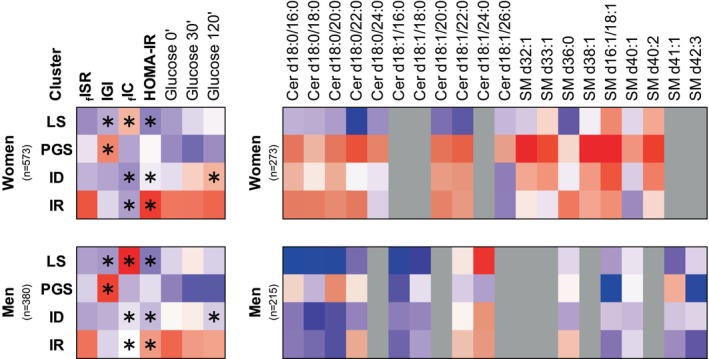
Cluster's profiling summary. Heatmap representing the scaled mean of each parameter. * in the left panel represents the significant differences (*p* < .05) in women vs. men within each cluster and for each parameter; detailed information on Table S2. In bold are parameters informing cluster analysis. Right Panel: Profile of lipid species that were significantly associated with the identified clusters in women and/or men. Only associations with *p* < .05 after Benjamini–Hochberg correction for multiple comparisons are displayed. Cluster names: LS, Liver‐sensitive; PGS, pancreas glucose sensitive; ID, insulin deficient; IR, insulin resistant. FISR, fasting insulin secretion rate; IGI, insulinogenic index; fIC, fasting insulin clearance; HOMA‐IR, Homeostatic Model Assessment for Insulin Resistance.

The IR cluster exhibited the most significant metabolic profile differences in glucose and lipid metabolism, impacting both sexes. This cluster was characterized by low insulin clearance, high insulin resistance, elevated Hepatic‐IR and Adipo‐IR, being more pronounced in women than in men (Figure 1C). Remarkably, women within this cluster displayed higher levels of DhCer (e.g.: Cer(d18:0/16:0), Cer(d18:0/18:0), Cer(d18:0/20:0) and Cer(d18:0/22:0); Figure 3), parallel to the pattern observed in the PGS cluster, indicating a heightened de novo ceramide synthesis in women compared to men. Furthermore, this cluster exhibited high SM36:0, already associated with liver disease progression,^23^ particularly in women. Besides, it also presented increased triglycerides (TGs), such as TG48:0, TG49:0 and TG50:0, mainly in women with normoglycemia. Certain detrimental lipid species, including TGs and CERs, were higher in women than in men, indicating a potential higher risk of cardiometabolic complications. Additionally, women in this cluster showed the highest levels of PC32:1 and PC34:1 despite the glycemic status, which could serve as an indicator of increased dysmetabolism risk in this subgroup.

## 4 DISCUSSION

This study sought to evaluate the association between insulin‐related mechanisms underlying diabetes, which differ by sex in conjunction with distinct glucose profiles. As insulin is lipogenic in nature, we examined lipid patterns based on sex differences to better identify individuals at risk for type 2 diabetes.

Using hierarchical cluster analysis, we analyzed insulin secretion, clearance and resistance in a cohort of 953 participants from the PREVADIAB2, previously enrolled in PREVADIAB1 without diabetes. We identified four distinct metabolic clusters: liver‐sensitive (LS), pancreas glucose‐sensitive (PGS), insulin‐deficient (ID) and insulin‐resistant (IR). These clusters exhibited varying dysglycemic risk: lowest in PGS and LS, highest in IR. PGS differs from LS due to higher insulin secretion, low insulin clearance, and increased hepatic insulin resistance. In a separate sex‐based analysis, metabolic patterns were consistent, but centroid differences emphasized sex‐related metabolic distinctions. This underscores the significance of sex‐related metabolic differences. Importantly, in our study, women showed higher BMI, IGI, hepatic‐IR and lower IC and ISI0‐120 than men, with lower fasting and 30‐min but higher 120‐min OGTT glucose. These results align with CA.ME.LI.A on fasting phenotypes^26^, ^27^ and, by adding OGTT data, confirm the DECODE pattern of greater IFG prevalence in men and IGT in women.^28^, ^29^ Our results reveal detectable glucose metabolism mechanistic changes leading to prediabetes/diabetes in normoglycemic subjects, highlighting the need for early proactive monitoring and intervention.

### 4.1 Clusters characterization

A novelty of our study is the lipidomic profiling conducted for each cluster separated by sex and adjusted for age, BMI and glycemia subgroups. Our clusters were derived with the primary aim of assessing insulin‐related mechanisms across the full spectrum of glucose tolerance—including individuals with normoglycemia, prediabetes and diabetes—unlike the approach of Ahlqvist et al., who examined only individuals with diabetes.^5^ This broader range enables the identification of early pathophysiological differences, whereas the Ahlqvist classification and subsequent refinements primarily describe heterogeneity within diagnosed diabetes. Furthermore, in contrast to our previous paper^6^ and Wagner et al.,^30^ who adjusted for sex but did not investigate sex‐specific pathophysiological mechanisms or incorporate lipidomics, our analysis integrated comprehensive lipidomics profiling and examined sex differences within clusters.

Previous works have explored the association between lipid species and metabolic disease,^31^, ^32^, ^33^ with conflicting findings, probably due to population diversity, namely regarding sex and age.

In our study, we observed distinct lipid patterns between men and women (Figures 2 and 3), with higher levels of specific circulating DhCer, CERs and SMs in women, indicating sex differences in lipid metabolism, while men had an increased CER/SM ratio. The metabolism of these sphingolipids is deeply connected, as they can be interconverted. Besides being important components of the cell membrane, sphingolipids can also act as messengers.^34^ Sphingolipid metabolism might impact insulin secretion and has been linked to insulin resistance.^21^, ^35^ Studies on mice lacking sphingomyelin synthase 1 revealed insulin secretion defects and a higher CER/SM ratio.^36^ CERs are associated with apoptosis, inflammation, and are used as biomarkers for dysmetabolism and cardiovascular conditions. Particularly, β‐cell apoptosis can be partially explained by the association between CERs and insulin deficiency. Interestingly, it has been shown that circulating CERs are originated mainly from the liver by de novo synthesis and are higher in subjects with MASLD.^9^, ^37^

Each cluster had a characteristic lipid signature consistent with its metabolic profile, reflecting unique combinations of glucose and blood lipids that may contribute to diabetes‐related complications.

#### 4.1.1 Liver Sensitive Cluster (LS)

The LS cluster exhibited the most favourable metabolic lipid signature, including higher insulin sensitivity and clearance compared to other clusters. The LS cluster displayed, indeed, the highest insulin clearance among all clusters, with hepatic insulin clearance being a key feature responsible for 50%–70% of whole‐body clearance. This may underlie its lower hepatic insulin resistance and favourable metabolic profile, as higher clearance is associated with lower insulin resistance states and reduced hepatic steatosis.^38^, ^39^ Such high clearance, influenced by genetic determinants and modulated by sex differences,^38^ could help protect against dysglycemia, whereas its impairment through environmental factors might contribute to diabetes development. This cluster displayed significantly lower levels of DhCer (e.g.: Cer(d18:0/16:0), Cer(d18:0/18:0), Cer(d18:0/20:0) and Cer(d18:0/22:0)), indicating reduced de novo ceramide synthesis. This cluster also displayed decreased levels of SM, suggesting limited ceramide accumulation through SM hydrolysis. Furthermore, the LS cluster demonstrated decreased Hepatic‐IR and NAFLD‐FLS scores, indicating reduced risk of non‐alcoholic fatty liver disease. The results also pointed to low DNL, as evidenced by the reduced levels of specific TGs, such as TG48:2 and TG56:4. Although these levels are slightly increased in individuals with dysglycemia within the LS cluster, they did not surpass those observed in other clusters, even among individuals with normoglycemia.

Notably, males in the LS cluster had higher levels of LPC18:2, a marker of enhanced insulin sensitivity.^40^ Overall, the LS profile represents a healthier metabolic state, highlighting the liver's role in maintaining metabolic balance and emphasizing insulin‐mediated processes in regulating glucose and liver functions.

#### 4.1.2 Pancreas Glucose Sensitive Cluster (PGS)

The PGS cluster exhibited a glycemic healthy profile, with all men being normoglycemic and a low proportion of dysglycemia in women. This cluster had the lowest glycemia in both sexes and fewer subjects progressing to dysglycemia, with the corresponding highest insulin secretion and disposition index. As the youngest group in our cohort, the PGS cluster showed the greatest pancreatic secretory capacity, reflected by a higher IGI, which helped preserve normoglycemia despite early signs of lipid profile compromise. Indeed, despite these positive aspects, subjects in this cluster showed a lipotoxic profile, mainly in women with dysglycemia, which presented a signature of higher levels of several DhCer (e.g., Cer(d18:0/16:0), Cer(d18:0/18:0), Cer(d18:0/20:0), Cer(d18:0/22:0)) and CER (Cer(d18:1/20:0), Cer(d18:1/22:0)), indicating increased de novo ceramide synthesis and accumulation. This lipid signature was linked to suppressed IC and Hepatic‐IR, which has been associated with liver steatosis, resulting in higher serum levels of DhCer and Cer.^41^, ^42^ The interplay between ceramides and insulin resistance is complex, with ceramides potentially inducing insulin resistance.^43^ Additionally, elevated sphingomyelins in this cluster may contribute to or be influenced by ceramide levels. This cluster also shows an increment in TGs, such as TG48:1, a marker of DNL^44^ associated with MASLD and increased risk of type 2 diabetes.^37^

These findings suggest that while a higher insulin secretion rate effectively impacts carbohydrate metabolism, it is also associated with liver insulin resistance, increased ceramide synthesis and enhanced DNL.

#### 4.1.3 Insulin Deficient Cluster (ID)

The ID cluster shows lower insulin secretion capacity with notable differences between women and men, paralleled to the pattern of the LS cluster, albeit with variations in levels and specific lipid species, especially concerning women. Surprisingly, women in this cluster displayed an increase in DhCer derived from CerS4 and CerS5/6 (DhCer (d18:0/20:0) and (d18:0/16:0)), indicating enhanced de novo ceramide synthesis compared to men, potentially influenced by low estradiol levels in menopause.^45^ In contrast, ID normoglycemic women exhibited the lowest levels of specific triglycerides (TG56:2, TG56:3, TG56:5 and TG56:7). These findings suggest that insulin secretion in this cluster is insufficient to support adequate glucose uptake. Interestingly, this impairment appears to have a lesser impact on hepatic lipid metabolism and markers of DNL.

#### 4.1.4 Insulin Resistant Cluster (IR)

In addition to aggravated glucose disturbances, the IR cluster exhibits a lipotoxic profile with elevated DhCer and ceramides, especially in women, suggesting increased de novo ceramide synthesis even in normoglycemia. Notably, the heightened ceramide levels display distinct species signatures between men (Cer(d18:1/24:0)) and women (Cer(d18:1/20:0) and Cer(d18:1/22:0)). Additionally, this profile is characterized by increased triglycerides (e.g., TG48:0, TG49:0, TG50:0) and decreased lysophosphatidylcholines (LPCs). This cluster, with the highest BMI, exhibits pronounced insulin resistance, exacerbated by reduced insulin clearance. Interestingly, reduced insulin clearance controlled by nitric oxide^46^ and is associated with lower hepatic carcinoembryonic antigen‐related cell adhesion molecule 1 (CEACAM1) levels, responsible for insulin receptor internalization, and declining human circulating CEACAM1 levels parallel the progression of insulin resistance, hyperinsulinemia, increased BMI and hepatic steatosis.^47^ Notably, the genetic regulation of IC by insulin‐degrading enzyme (IDE) is compromised as prediabetes advances.^38^ The loss of hepatic IDE not only raises glucose levels but also increases hepatic triglyceride accumulation.^38^ Our study observed that women's higher TG/ceramide levels may indicate a higher peripheral insulin resistance when related to IGT pathway, while men's when they have an IFG profile concomitant with hepatic/visceral adiposity may confer earlier macrovascular risk, as suggested by the CA.ME.LI.A study.^26^

High DhCer have been linked to insulin resistance and MASLD.^9^, ^37^ Specifically, increased levels of DhCer(d18:0/22:0) and Cer(d18:1/18:0), Cer(d18:1/20:0) and Cer(d18:1/22:0) were associated with increased susceptibility to type 2 diabetes^48^ and DhCer C18:0 was elevated in human plasma samples 9 years before developing type 2 diabetes^13^ suggesting a potential ceramide association with baseline glucose levels.^49^

Additionally, IR women exhibit significantly higher levels of PC32:1 and PC34:1, which contain myristic and palmitic acids derived from DNL or circulation. PC32:1 elevation in normoglycemic women indicates it as a marker for dysmetabolism risk,^50^ highlighting the liver's role in glucose and lipid regulation via insulin‐mediated mechanisms, impacting metabolic pathways.

In conclusion, women exhibit increased DhCer and Cer levels, particularly in the PGS and IR clusters, compared to men, indicating a higher cardiometabolic risk (Figure 3). Notably, a better lipid profile is associated only with low hepatic insulin resistance, indicating its relevance. C16:0 and C18:0 dihydroceramides have been previously related to insulin resistance in other studies profile,^51^ independent of BMI and visceral adipose tissue. These species have been suggested to be related to a metabolically healthier profile,^51^ with lower body fat deposition. Interestingly, ultralong chain ceramides (>C26) seem to be high in PGS women with normoglycemia but not with dysglycemia.

Men additionally had a higher LPC to PC ratio; specifically, LPC18:0 and LPC18:2 were higher in men in the LS and ID clusters with normoglycemia, decreasing in hyperglycemia. This finding aligns with a previous study where LPC18:2 was inversely related to progression to type 2 diabetes.^10^ Also, it has been suggested that LPC18:0 may decrease glycemia through PPAR gamma activity.^9^

The lipid signature of these clusters corresponds with their metabolic profiles, showing distinct combinations between glucose and blood lipids that we postulate to be responsible, along with other factors, for diabetes complications.

This study has limitations, notably its focus on an aging population and the inability to determine whether the observed patterns are genetically driven, shaped by lifestyle or a combination of both, and the lack of information on prior or ongoing dietary control. In the trajectory to real‐world application of the present study, we acknowledge that our clusters were derived from biochemical and metabolic indexes not routinely measured in clinical practice, beyond the HOMA‐IR; yet parameters such as IGI, _f_ISR and insulin clearance, while less common, could be feasibly incorporated into routine workflows with appropriate informatics support.

Our findings underscore the importance of viewing the path to diabetes through a broader metabolic lens—beyond glycemia—by integrating sex differences and lipidomic signatures. Notably, distinct metabolic clusters identified in this study reflect varying degrees of risk even before diabetes onset, suggesting that metabolic heterogeneity precedes current diagnostic thresholds. Understanding these cluster‐specific profiles—including lipid signatures—may enable more precise, individualized strategies for diabetes prevention and early intervention, advancing the goals of precision medicine.

## AUTHOR CONTRIBUTIONS

Conceptualization: L.G.‐C., J.M.B., J.F.R. and M.P.M; Methodology: A.G., M.P.M., M.J.M, A.F.P., B.P. and F.C.; Software: A.F.P. and M.J.M.; Formal analysis: A.F.P., M.J.M. and F.C.; Discussion: A.F.P., M.J.M, A.G. and M.P.M.; Writing—original draft preparation: A.F.P., M.J.M, A.G. and M.P.M.; Writing—review and editing: all authors; Funding acquisition: M.P.M. and A.G. All authors approved the final version of the manuscript. M.P.M. is the guarantor of this work and, as such, had full access to all the data in the study and takes responsibility for the integrity of the data and the accuracy of the data analysis.

## CONFLICT OF INTEREST STATEMENT

The authors declare no conflict of interest regarding the content of this article.

## Supporting information


Data S1.

## Data Availability

The data that support the findings of this study are available upon reasonable request to the corresponding author. The data are not publicly available due to privacy and ethical restrictions.
