# Supplementary material for: Lipidomic profiling unveils sex differences in diabetes risk: Implications for precision medicine
Source: Eur J Clin Invest. 2025 Oct 24;56(1):e70137. doi: 10.1111/eci.70137 (PMC12817243; doi:10.1111/eci.70137)
Supplement: Supplementary file 1 — Data S1. [file ECI-56-e70137-s001.docx]

Appendix S1

Methods

*Clinical and Biochemical Parameters*

The clinical history of participants in the PREVADIAB2 study was reviewed, with particular attention to their use of anti-diabetic medications. Body weight was recorded using an electronic scale with a precision of 0.01 kg, while participants wore minimal clothing and were barefoot. Waist circumference was measured with a flexible tape placed between the upper part of the iliac crest and the bottom edge of the ribs. Height was measured to the nearest 0.1 cm using a stadiometer, following WHO standardized guidelines. Blood samples were drawn from the antecubital vein after an overnight fast, with participants seated and at rest. These samples were taken at three time points: at baseline, 30 minutes, and 120 minutes into the oral glucose tolerance test (OGTT). Blood was collected into both dry tubes and tubes containing EDTA as an anticoagulant. Afterward, the biological samples were centrifuged at 3500 rpm for 10 minutes at a temperature of 4°C, and the plasma and serum were frozen at −80°C for future analysis.

Serum samples were used to assess the participants' lipid profiles (e.g.: total cholesterol, LDL-c, HDL-c, free fatty acids, and triglycerides). Glycated hemoglobin (HbA1c) was measured using high-performance liquid chromatography (HPLC) with boronate affinity (Menarini Premier Hb 9210). Plasma glucose levels were determined using a glucose analyzer based on the glucose oxidase method (Olympus AU640, Beckman Coulter, Brea, CA, USA), while plasma insulin and C-peptide concentrations were measured using chemiluminescence assays (Liaison, DiaSorin, Salugia, Piedmont, Italy).

Supplementary Figure S1. Methodology Flowchart. In 2014, 1088 subjects from the PREVADIAB1 study without type 2 diabetes in 2008–2009 were requested to participate in the follow-up study – PREVADIAB 2 cohort. After the application of exclusion criteria and preprocessing the data, we performed a cluster analysis where individuals were separated by sex. We profiled the clusters with several metabolic parameters. Finally, we performed a lipidomic analysis on a stratified sampling procedure across clusters and gender strata of 488 individuals to further profiling the clusters, overall and separated by sex.

Supplementary Figure S2. PCA analyses of samples grouped by clusters before (A) and after (B) outliers’ exclusion.

**
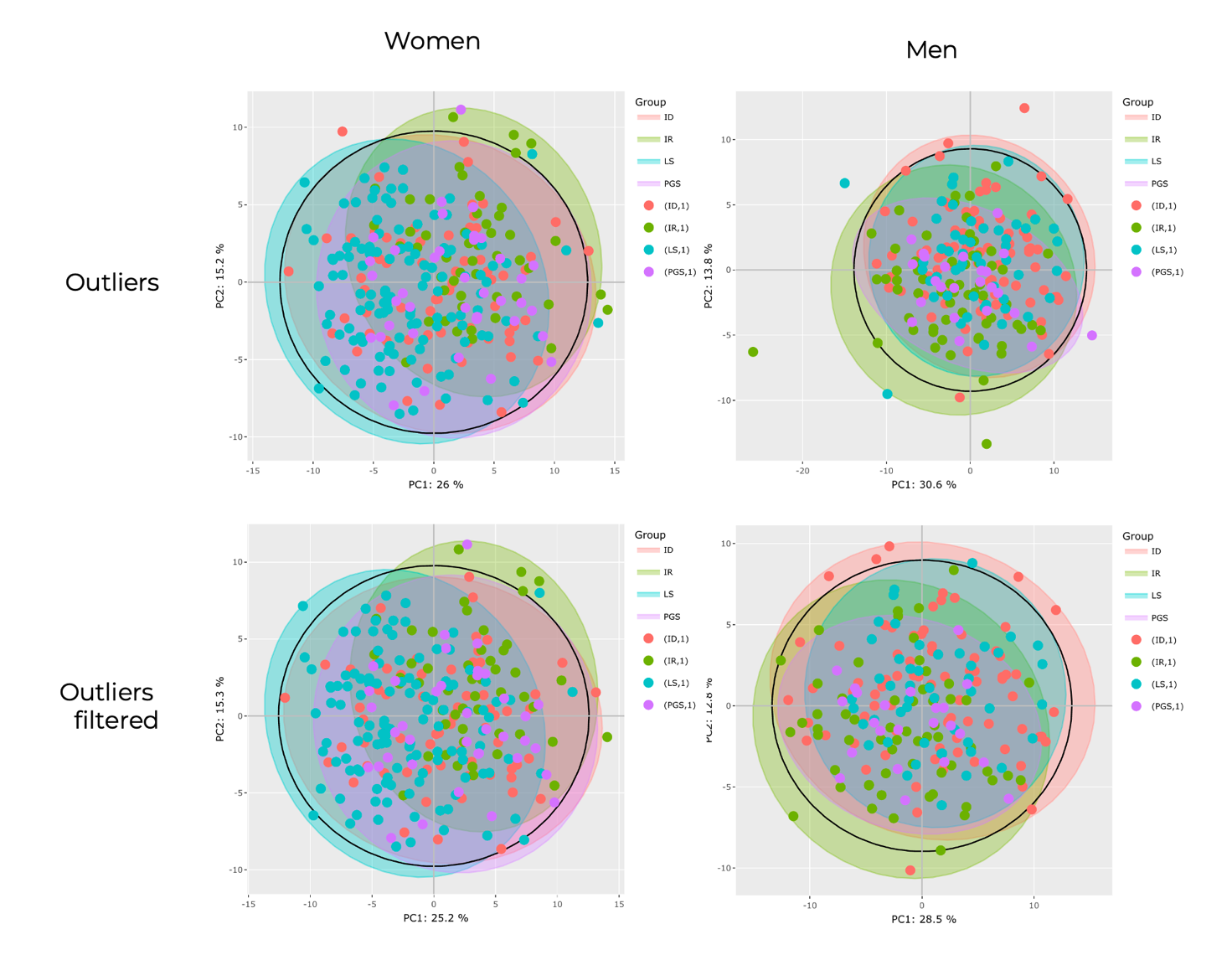
**

**Supplementary Figure S3. Cluster's profile by sex.** Heatmap representing the scaled mean of each parameter by sex. A) Normoglycemia subgroup; B) Dysglycemia subgroup includes prediabetes and diabetes. Cluster names: liver-sensitive (LS); pancreas glucose sensitive (PGS); insulin deficient (ID); insulin resistance (IR).

Supplementary Figure S4. Clusters' lipidomic profiling scaled by the overall population. Heatmap representing the scaled mean of each parameter for the entire cohort. CER – Ceramides; ID – Insulin deficient; IR – Insulin resistant, LS – Liver sensitive; LysoPC – Lysophosphatidylcholine; PC – Phosphatidylcholine; SM – Sphingomyelin; PGS – Pancreas glucose sensitive; TG – Triglycerides.

**Men**

**Women**


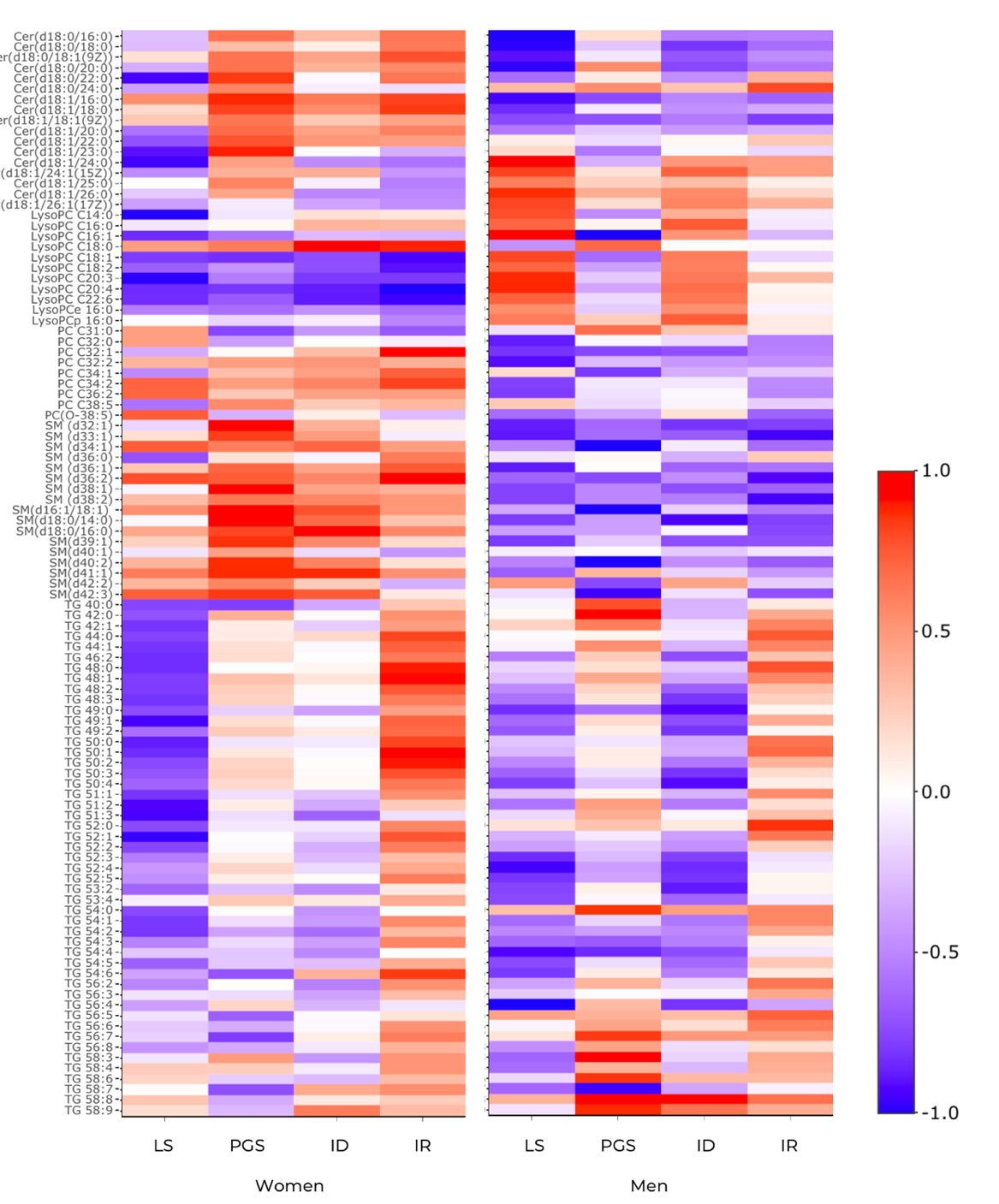


|  |
| --- |
| Cer (d18:0/16:0) |
| Cer (d18:0/18:0) |
| Cer (d18:0/18:1(9Z)) |
| Cer (d18:0/20:0) |
| Cer (d18:0/22:0) |
| Cer (d18:0/24:0) |
| Cer (d18:1/16:0) |
| Cer (d18:1/18:0) |
| Cer (d18:1/18:1(92) |
| Cer (d18:1/20:0) |
| Cer (d18:1/22:0) |
| Cer (d18:1/23:0) |
| Cer (d18:1/24:0) |
| Cer (d18:1/24:1(15Z)) |
| Cer (d18:1/25:0) |
| Cer (d18:1/26:0) |
| Cer (d18:1/26:1(17Z)) |
| LysoPC C14:0 |
| LysoPC C16:0 |
| LysoPC C16:1 |
| LysoPC C18:0 |
| LysoPC C18:1 |
| LysoPC C18:2 |
| LysoPC C20:3 |
| LysoPC C20:4 |
| LysoPC C22:6 |
| LysoPCe 16:0 |
| LysoPCp 16:0 |
| PC C31:0 |
| PC C32:0 |
| PC C32:1 |
| PC C32:2 |
| PC C34:1 |
| PC C34:2 |
| PC C36:2 |
| PC C38:5 |
| PC (O-38:5) |
| SM (d32:1) |
| SM (d33:1) |
| SM (d34:1) |
| SM (d36:0) |
| SM (d36:1) |
| SM (d36:2) |
| SM (d38:1) |
| SM (d38:2) |
| SM (d16:1/18:1) |
| SM (d18:0/14:0) |
| SM (d18:0/16:0) |
| SM (d39:1) |
| SM (d40:1) |
| SM (d40:2) |
| SM (d41:1) |
| SM (d42:2) |
| SM (d42:3) |
| TG 40:0 |
| TG 42:0 |
| TG 42:1 |
| TG 44:0 |
| TG 44:1 |
| TG 46:2 |
| TG 48:0 |
| TG 48:1 |
| TG 48:2 |
| TG 48:3 |
| TG 49:0 |
| TG 49:1 |
| TG 49:2 |
| TG 50:0 |
| TG 50:1 |
| TG 50:2 |
| TG 50:3 |
| TG 50:4 |
| TG 51:1 |
| TG 51:2 |
| TG 51:3 |
| TG 52:0 |
| TG 52:1 |
| TG 52:2 |
| TG 52:3 |
| TG 52:4 |
| TG 52:5 |
| TG 53:2 |
| TG 53:4 |
| TG 54:0 |
| TG 54:1 |
| TG 54:2 |
| TG 54:3 |
| TG 54:4 |
| TG 54:5 |
| TG 54:6 |
| TG 56:2 |
| TG 56:3 |
| TG 56:4 |
| TG 56:5 |
| TG 56:6 |
| TG 56:7 |
| TG 56:8 |
| TG 58:3 |
| TG 58:4 |
| TG 58:6 |
| TG 58:7 |
| TG 58:8 |
| TG 58:9 |

Cluster

N

60

84

23

48

119

38

37

50

**Supplementary Figure S5. Lipidomics cluster's profiling.** A-B – Heatmap representing the scaled mean of each lipid specie, divided by normoglycemia (A) and dysglycemia (PD and Diabetes; B). Lipid Species: CER – ceramides; LysoPC – lysophosphatidylcholine; PC – phosphatidylcholine; SM – sphingomyelin; TG – triglycerides. Clusters Names: ID – insulin deficient; IR – insulin resistant, LS – liver sensitive; PGS – pancreas glucose sensitive. NS – non-significant.


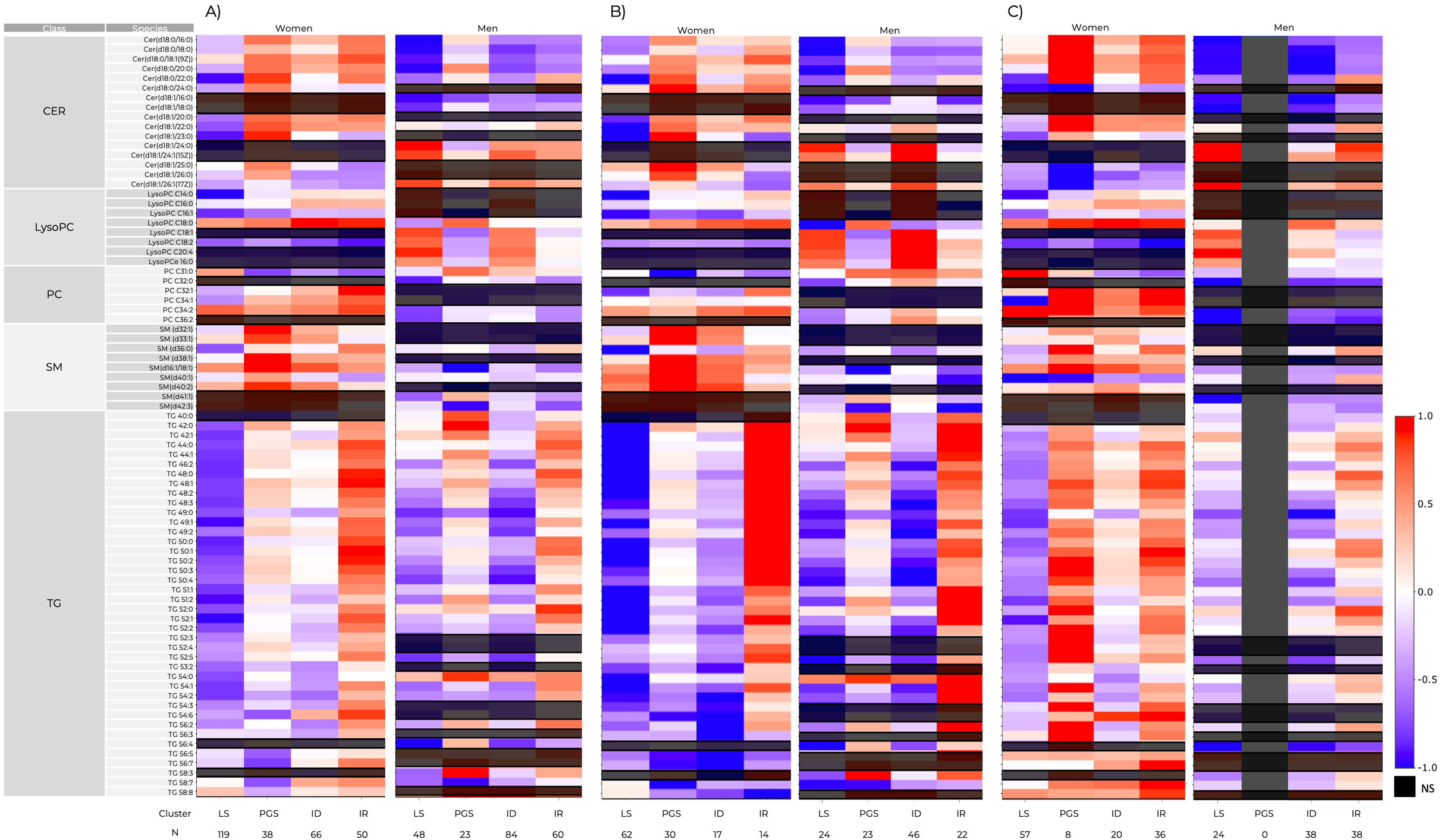


**C**

**B**

**A**


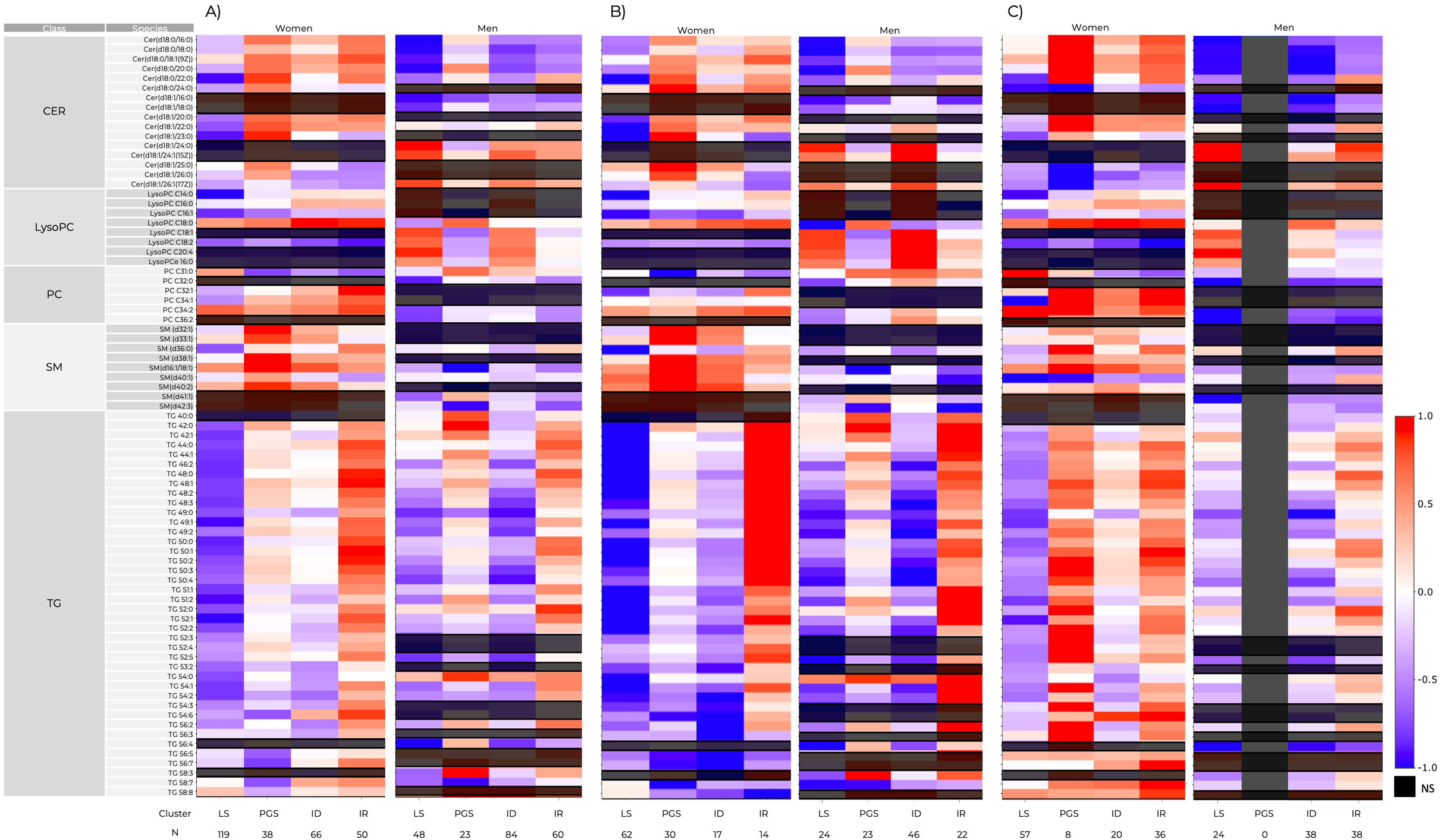


**Dysglycemia**

**Normoglycemia**

**Supplementary Table S1.** Variable importance for cluster analysis. Variable importance in projection (VIP) values of the parameters informing the cluster analysis: of the overall population as well as for both sexes separately. IC – Insulin clearance; IGI – Insulinogenic index; ISR – Insulin secretion rate; f denotes fast.

| VIP overall population | VIP women | VIP men |
| --- | --- | --- |
| IGI  (1.1) | _f_IC  (1.1) | _f_IC  (1.2) |
| _f_IC  (1.0) | HOMA-IR  (1.1) | IGI  (1.0) |
| HOMA-IR  (1.0) | IGI  (1.0) | _f_ISR  (1.0) |
| _f_ISR  (0.9) | _f_ISR  (0.8) | HOMA-IR  (0.8) |

Supplementary Table S2. Summary statistics of Clusters for the cluster analysis including the overall population. Parameters are reported for overall population and for women and men. In bold are the parameters informing cluster analysis. Values are reported as mean±SD for continuous parameters and as count and percentages for categorical parameters. P-values are reported for comparison between sex, performed with Mann–Whitney test for continuous variables and with chi-square test for categorical variables. Abbreviations: BMI, body mass index; fISR, fasting insulin secretion rate; IGI, insulinogenic index; fIC, fasting insulin clearance; HOMA-IR, Homeostatic Model Assessment for Insulin Resistance; ISI, insulin sensitivity index; Adipo-IR, adipose tissue insulin resistance index; Hepatic-IR, hepatic insulin resistance index; DI, disposition index; NAFLD-FLS, non-alcoholic fatty liver disease-fatty liver score.

|  | **LS** | | | **PGS** | | | **ID** | | | **IR** | | |
| --- | --- | --- | --- | --- | --- | --- | --- | --- | --- | --- | --- | --- |
|  | Women | Men | Men vs  Women  (P-value) | Women | Men | Men vs  Women  (P-value) | Women | Men | Men vs  Women  (P-value) | Women | Men | Men vs  Women  (P-value) |
| Total, n (%) | 285 (50) | 98 (26) | - | 80 (14) | 24 (6) | - | 146 (25) | 173 (46) | - | 62 (11) | 85 (22) | - |
| Normoglycemia, n (%)  Prediabetes, n (%)  Diabetes, n (%) | 228 (80)  45 (16)  12 (4) | 72(74)  21(21)  5(5) | 0.08 | 72 (90)  7 (9)  1 (1) | 24 (100)  0 (0)  0 (0) | 0.5 | 96 (66)  45 (31)  5 (3) | 135 (78)  33 (19)  5 (3) | 0.02* | 25 (40)  28 (45)  9 (15) | 45 (53)  29 (34)  11 (13) | 0.6 |
| Age, Years | 62±13 | 62±11 | 0.8 | 55±13 | 56±14 | 0.8 | 61±12 | 60±14 | 0.6 | 63±12 | 62±13 | 0.7 |
| BMI, Kg/m^2^ | 27±4.2 | 26±3 | 0.3 | 28±5 | 26±3 | 0.17 | 28±4 | 26±3 | <0.001* | 32±4 | 30±4 | <0.001* |
| **Fast-ISR, pmol/min** | 131±38 | 142±47 | 0.05 | 181±57 | 170±53 | 0.5 | 156±49 | 143±41 | 0.01 | 309±75 | 297±82 | 0.2 |
| **IGI** | 0.68±0.44 | 0.46±0.34 | <0.001* | 2.3±1.3 | 3.1±1.8 | 0.005* | 0.65±0.29 | 0.70±0.4 | 0.7 | 1.01±0.68 | 0.97±0.83 | 0.3 |
| **Fast-IC, L/min** | 4.9±1.1 | 6.8±1.5 | <0.001* | 3.3±1.1 | 3.3±0.5 | 0.1 | 2.9±0.6 | 3.8±0.9 | <0.001* | 3.2±0.96 | 4.1±1.0 | <0.001* |
| **HOMA-IR** | 1.0±0.35 | 0.81±0.34 | <0.001* | 2.16±0.85 | 1.9±0.7 | 0.2 | 1.98±0.63 | 1.55±0.72 | <0.001* | 4.2±1.2 | 3.2±1.2 | <0.001* |
| ISI_0-120_, Log10 | 73±16 | 81±22 | <0.001* | 69±20 | 73±16 | 0.2 | 60±14 | 70±17 | <0.001* | 46±10 | 53±14 | 0.001* |
| Adipo-IR | 16±8 | 12±7 | <0.001* | 32±19 | 24±14 | 0.06 | 32±15 | 25±14 | <0.001* | 58±23 | 44±21 | <0.001* |
| Hepatic-IR, x10^4^ | 5.2±2.8 | 4.3±3.9 | <0.001* | 1.1±6.6 | 1.2±8.2 | 0.9 | 6.8±3.2 | 6.4±3.6 | 0.09 | 12.9±7.2 | 11.0±8.2 | 0.005* |
| Disposition Index, mmol^-1^ | 2.6±1.9 | 2.4±1.9 | 0.08 | 5.0±4.9 | 7±5.3 | 0.005* | 1.4±0.8 | 2.0±1.4 | <0.001* | 0.99±0.54 | 1.2±0.8 | 0.07 |
| NAFLD-FLS | -1.9±0.83 | -2.0±0.90 | 0.2 | -0.96±1.1 | -1.3±1.0 | 0.2 | -1.1±0.90 | -1.4±1.05 | <0.001* | 0.80±0.96 | 0.27±1.03 | 0.003* |
| Glucose 0 min, mmol/L | 5±0.6 | 5.1±0.7 | 0.2 | 4.9±0.5 | 4.9±0.4 | 0.7 | 5.1±0.6 | 5.2±0.6 | 0.3 | 5.6±0.7 | 5.7±1.0 | 0.8 |
| Glucose 30 min, mmol/L | 8.3±1.6 | 8.7±1.8 | 0.09 | 7.2±1.4 | 6.8±1.4 | 0.18 | 9.0±1.5 | 8.7±1.6 | 0.1 | 10.0±1.8 | 9.7±2.3 | 0.07 |
| Glucose 120 min, mmol/L | 6.3±1.9 | 6.1±2.4 | 0.06 | 5.6±1.6 | 4.9±1.1 | 0.05 | 7.1±2.0 | 6.1±1.9 | <0.001* | 7.9±2.4 | 7.3±3.1 | 0.05 |
| Total cholesterol, mg/dL | 203±33.9 | 196±42.7 | 0.06 | 209±35.3 | 196±34.2 | 0.3 | 203±37.9 | 198±38.3 | 0.4 | 200±37.2 | 194±38.2 | 0.4 |
| LDL-c, mg/dL | 137±28.5 | 133±35.4 | 0.2 | 145±29.6 | 140±31.6 | 0.7 | 137±30.7 | 138±31.2 | 0.7 | 140±30.5 | 137±30.3 | 0.7 |
| HDL-c, mg/dL | 57.5±11.3 | 52.0±11.3 | <0.001* | 54.6±10.3 | 46.7±8.51 | <0.001* | 57.5±13.9 | 51.2±11.4 | <0.001* | 46.8±9.40 | 43.3±10.1 | 0.02 |
| Triglycerides, mg/dL | 98.1±43.4 | 122±66.4 | <0.001* | 117±49.5 | 115±36.6 | 0.8 | 117±58.7 | 107±53.5 | 0.06 | 161±59.3 | 164±87.4 | 0.3 |

**Supplementary Table S3. Summary statistics of the clusters profiled by subgroups of normoglycemia and dysglycemia.** Values are reported as mean±sd. P-values are reported for comparison between subgroups, performed with Mann–Whitney test with Bonferroni correction. IC - Insulin Clearance; ID – Insulin deficient; IR – Insulin resistant; ISR – Insulin Secretion Rate; LS – Liver sensitive; PGS – Pancreas glucose sensitive. ns – non-significant (p>0.05). *As there are no men with dysglycemia in the PGS cluster, results for this cluster are only regarding women.*

|  | **LS** | | | | | | **PGS** | | | **ID** | | | | | | **IR** | | | | | |
| --- | --- | --- | --- | --- | --- | --- | --- | --- | --- | --- | --- | --- | --- | --- | --- | --- | --- | --- | --- | --- | --- |
|  | Women Normo | Women Dysg | P -value | Men Normo | Men Dysg | P -value | Women Normo | Women Dysg | P -value | Women Normo | Women Dysg | P -value | Men Normo | Men Dysg | P -value | Women Normo | Women Dysg | P -value | Men Normo | Men Dysg | P -value |
| N | 228 | 57 | - | 72 | 26 | - | 72 | 8 | - | 96 | 50 | - | 135 | 38 | - | 25 | 37 | - | 45 | 40 | - |
| Age, Years | 60±12 | 69±7 | <0.001 | 64±10 | 67±9 | ns | 58±13 | 54±16 | ns | 56±11 | 65±11 | 0.05 | 60±14 | 67±11 | ns | 54±16 | 65±10 | ns | 55±16 | 67±8 | 0.003 |
| BMI, Kg/m² | 27±5 | 27±4.0 | ns | 26±4 | 26±3 | ns | 27±5 | 31±3 | ns | 28±6 | 28±4 | ns | 25±2 | 27±3 | 0.003 | 34±3 | 33±4 | ns | 31±3 | 30±3 | ns |
| **Fast-ISR, pmol/min** | 135±43 | 138±35 | ns | 147±50 | 144±31 | ns | 186±58 | 220±44 | ns | 162±53 | 165±45 | ns | 132±45 | 166±39 | 0.006 | 327±96 | 313±75 | ns | 295±77 | 305±86 | ns |
| **IGI** | 0.79±0.50 | 0.37±0.26 | <0.001 | 0.58±0.43 | 0.27±0.16 | 0.02 | 2.74±1.81 | 1.54±0.71 | ns | 0.69±0.25 | 0.49±0.22 | 0.05 | 0.67±0.36 | 0.53±0.34 | ns | 1.5±0.77 | 0.81±0.44 | 0.005 | 1.15±0.89 | 0.94±1 | ns |
| **Fast-IC, L/min** | 4.9±1.0 | 5.0±1.2 | ns | 6.5±1.0 | 6.4±1.0 | ns | 3.5±1.0 | 2.9±1.1 | ns | 3.1±0.5 | 3.0±0.6 | ns | 4.1±0.8 | 3.8±0.8 | ns | 3.1±0.9 | 3.2±1.0 | ns | 4.1±1.0 | 4.1±1.1 | ns |
| **HOMA-IR** | 1.0±0.39 | 1.1±0.36 | ns | 0.85±0.32 | 0.95±0.31 | ns | 2.1±0.86 | 2.9±0.61 | ns | 2.0±0.7 | 2.2±0.58 | ns | 1.24±0.5 | 1.9±0.79 | <0.001 | 4.3±1.33 | 4.3±1.16 | ns | 2.91±1.04 | 3.57±1.20 | ns |
| ISI_0-120_, Log10 | 77±15 | 58±7 | <0.001 | 89±21 | 63±16 | <0.001 | 71±21 | 43±5 | <0.001 | 62±14 | 49±9 | <0.001 | 78±18 | 53±9 | <0.001 | 52±13 | 40±6 | 0.005 | 60±16 | 45±8 | <0.001 |
| Adipo-IR | 16±9 | 21±10 | 0.05 | 13±8 | 16±7 | ns | 28±17 | 62±14 | <0.001 | 30±17 | 39±16 | ns | 19±10 | 35±14 | <0.001 | 55±27 | 63±21 | ns | 42±27 | 49±21 | ns |
| Hepatic-IR, x10^4^ | 5.6±2.9 | 4.0±2.1 | 0.04 | 4.7±4.2 | 3.3±2.0 | ns | 11.8±7.7 | 16.8±3.0 | ns | 7.6±3.6 | 6.6±3.6 | ns | 5.5±3.4 | 6.3±3.7 | ns | 15.7±9.5 | 13.0±6.9 | ns | 12.8±7.8 | 10.9±9.8 | ns |
| Disposition Index, mmol^-1^ | 2.9±1.9 | 1.3±0.9 | <0.001 | 2.6±1.8 | 1.2±0.6 | 0.02 | 6.1±6.2 | 2.1±1.3 | 0.01 | 1.5±0.9 | 0.9±0.5 | ns | 2.3±1.3 | 1.3±0.9 | <0.001 | 1.3±0.5 | 0.8±0.3 | 0.003 | 1.5±0.7 | 1.1±1.0 | ns |
| NAFLD-FLS | -2.0±0.81 | -1.4±0.81 | 0.006 | -2.0±0.73 | -1.5±0.84 | ns | -1.1±0.96 | -0.1±1.2 | ns | -1.2±0.96 | -0.8±0.83 | ns | -1.8±0.98 | -1.0±1.01 | 0.005 | 0.96±1.031 | 0.88±0.98 | ns | 0.27±1.2 | 0.53±1.01 | ns |
| Glucose 0 min, mmol/L | 4.9±0.48 | 5.4±0.7 | <0.001 | 4.9±0.55 | 5.7±0.84 | <0.001 | 4.9±0.47 | 5.1±0.61 | ns | 4.9±0.51 | 5.5±0.73 | <0.001 | 5.1±0.45 | 5.7±0.85 | 0.002 | 5.3±0.51 | 5.8±0.76 | 0.08 | 5.2±0.52 | 6.3±0.98 | <0.001 |
| Glucose 30 min, mmol/L | 8.0±1.46 | 9.5±1.38 | <0.001 | 8.3±1.47 | 9.9±2.0 | 0.002 | 7.0±1.29 | 9.23±1.24 | 0.003 | 8.5±1.35 | 9.9±1.41 | <0.001 | 8.4±1.36 | 9.9±1.91 | <0.001 | 9.0±1.61 | 10.7±1.50 | <0.001 | 8.8±1.53 | 10.6±2.66 | 0.03 |
| Glucose 120 min, mmol/L | 5.6±1.16 | 9.3±1.57 | <0.001 | 5.0±1.31 | 9.1±2.24 | <0.001 | 5.2±1.07 | 8.9±1.60 | <0.001 | 6.0±1.13 | 9.2±1.72 | <0.001 | 5.4±1.20 | 8.3±2.18 | <0.001 | 6.2±1.0 | 9.1±2.45 | <0.001 | 5.4±1.13 | 9.6±3.0 | <0.001 |

Supplementary Table S4. P-values for association of lipid species within the clusters in each sex group adjusted by age, BMI and glycemic class (Normoglycemia and dysglycemia). P-values are also adjusted for multicomparison (Benjamini-Hochberg). Colors represent lipid species that are associated with women, men or both. Lipidomic analysis was performed in 273 women and 215 men, please refer to the materials and methods for more detailed information. CER – Ceramides; LysoPC – Lysophosphatidylcholine; PC – Phosphatidylcholine; SM - Sphingomyelin; TG – Triglycerides.

| **Species** | **Women** | **Men** |  | **Species** | **Women** | **Men** |
| --- | --- | --- | --- | --- | --- | --- |
| Cer (d18:0/24:0) | 1,59E-03 | 6,05E-02 |  | TG 42:1 | 1,31E-04 | 2,78E-03 |
| Cer (d18:1/20:0) | 3,04E-03 | 6,91E-02 |  | TG 44:0 | 1,66E-09 | 6,63E-03 |
| Cer (d18:1/23:0) | 1,39E-02 | 9,73E-02 |  | TG 44:1 | 9,66E-09 | 6,51E-04 |
| Cer (d18:1/25:0) | 1,44E-03 | 1,31E-01 |  | TG 46:2 | 1,31E-10 | 4,76E-05 |
| Cer (d18:1/26:0) | 1,10E-03 | 1,02E-01 |  | TG 48:0 | 3,04E-14 | 1,70E-06 |
| LysoPC C14:0 | 3,45E-04 | 2,55E-01 |  | TG 48:1 | 3,04E-14 | 4,76E-05 |
| LysoPC C16:0 | 3,69E-04 | 7,40E-01 |  | TG 48:2 | 2,75E-12 | 8,06E-05 |
| LysoPC C16:1 | 1,49E-02 | 1,31E-01 |  | TG 48:3 | 4,86E-10 | 1,63E-05 |
| PC C32:1 | 5,24E-06 | 4,69E-01 |  | TG 49:0 | 5,24E-06 | 4,76E-05 |
| PC C34:1 | 1,84E-02 | 9,95E-02 |  | TG 49:1 | 1,31E-10 | 1,44E-04 |
| SM (d32:1) | 2,33E-02 | 8,71E-01 |  | TG 49:2 | 4,22E-10 | 1,12E-03 |
| SM (d33:1) | 2,53E-02 | 4,93E-01 |  | TG 50:0 | 4,43E-13 | 5,68E-07 |
| SM (d38:1) | 1,07E-02 | 6,84E-01 |  | TG 50:1 | 1,11E-16 | 2,47E-05 |
| SM (d40:2) | 2,76E-02 | 5,09E-01 |  | TG 50:2 | 5,70E-14 | 6,51E-04 |
| TG 52:3 | 8,67E-03 | 2,19E-01 |  | TG 50:3 | 2,75E-12 | 8,98E-04 |
| TG 52:4 | 2,22E-02 | 9,95E-02 |  | TG 50:4 | 2,92E-09 | 2,95E-04 |
| TG 53:2 | 3,93E-02 | 8,21E-02 |  | TG 51:1 | 1,58E-10 | 2,18E-03 |
| TG 54:3 | 6,45E-03 | 4,98E-01 |  | TG 51:2 | 2,37E-04 | 3,94E-02 |
| TG 54:6 | 9,89E-05 | 9,95E-02 |  | TG 52:0 | 4,43E-13 | 1,63E-05 |
| TG 56:3 | 1,00E-02 | 3,41E-01 |  | TG 52:1 | 3,32E-11 | 5,52E-06 |
| TG 56:5 | 2,53E-02 | 7,97E-01 |  | TG 52:2 | 3,90E-07 | 2,74E-02 |
| TG 56:7 | 1,37E-03 | 8,95E-01 |  | TG 52:5 | 5,78E-04 | 1,60E-02 |
| TG 58:8 | 1,28E-03 | 9,73E-02 |  | TG 54:0 | 1,44E-04 | 4,76E-05 |
| Cer (d18:1/16:0) | 1,84E+01 | 3,96E-02 |  | TG 54:1 | 5,75E-12 | 5,68E-07 |
| Cer (d18:1/18:0) | 7,51E-02 | 4,57E-02 |  | TG 54:2 | 6,21E-07 | 1,00E-02 |
| Cer (d18:1/24:0) | 6,17E-02 | 3,58E-02 |  | TG 56:2 | 2,57E-05 | 1,89E-03 |
| Cer (d18:1/24:1(15Z)) | 5,67E-02 | 1,64E-02 |  | TG 58:7 | 1,59E-03 | 2,23E-02 |
| LysoPC C18:1 | 1,56E-01 | 1,00E-02 |  | Cer (d18:1/18:1(9Z)) | 2,91E-01 | 4,69E-01 |
| LysoPC C20:4 | 8,18E-01 | 4,37E-02 |  | LysoPC C20:3 | 1,85E-01 | 2,65E-01 |
| LysoPCe 16:0 | 1,34E-01 | 1,25E-02 |  | LysoPC C22:6 | 7,20E-01 | 3,32E-01 |
| PC C32:0 | 5,67E-02 | 3,03E-02 |  | LysoPCp 16:0 | 1,44E-01 | 3,10E-01 |
| PC C36:2 | 3,05E-01 | 1,18E-02 |  | PC C32:2 | 9,03E-02 | 1,02E-01 |
| SM (d41:1) | 1,60E-01 | 3,63E-02 |  | PC C38:5 | 9,92E-02 | 5,10E-01 |
| SM (d42:3) | 1,33E-01 | 1,81E-02 |  | PC (0-38:5) | 1,15E-01 | 2,55E-01 |
| TG 40:0 | 1,44E-01 | 1,16E-02 |  | SM (d34:1) | 6,26E-01 | 2,88E-01 |
| TG 56:4 | 3,05E-01 | 4,35E-02 |  | SM (d36:1) | 6,27E-02 | 2,19E-01 |
| TG 58:3 | 1,63E-01 | 3,98E-03 |  | SM (d36:2) | 7,51E-02 | 4,02E-01 |
| Cer (d18:0/16:0) | 3,53E-07 | 2,23E-02 |  | SM (d38:2) | 1,57E-01 | 9,95E-02 |
| Cer (d18:0/18:0) | 5,94E-13 | 4,43E-02 |  | SM (d18:0/14:0) | 6,12E-02 | 6,95E-01 |
| Cer (d18:0/18:1(9Z)) | 1,22E-03 | 1,18E-02 |  | SM (d18:0/16:0) | 1,84E-01 | 1,30E-01 |
| Cer (d18:0/20:0) | 1,62E-08 | 4,76E-05 |  | SM (d39:1) | 8,60E-02 | 6,45E-01 |
| Cer (d18:0/22:0) | 4,94E-05 | 2,08E-02 |  | SM (d42:2) | 3,63E-01 | 1,12E-01 |
| Cer (d18:1/22:0) | 1,29E-04 | 1,64E-02 |  | TG 51:3 | 5,67E-02 | 4,26E-01 |
| Cer (d18:1/26:1(17Z)) | 1,56E-02 | 1,48E-02 |  | TG 53:4 | 3,09E-01 | 2,99E-01 |
| LysoPC C18:0 | 4,48E-02 | 4,18E-02 |  | TG 54:4 | 6,62E-01 | 5,09E-01 |
| LysoPC C18:2 | 8,21E-04 | 1,64E-02 |  | TG 54:5 | 6,39E-02 | 1,28E-01 |
| PC C31:0 | 1,10E-03 | 2,96E-03 |  | TG 56:6 | 6,17E-02 | 6,13E-01 |
| PC C34:2 | 3,41E-02 | 2,42E-02 |  | TG 56:8 | 5,67E-02 | 5,84E-01 |
| SM (d36:0) | 3,35E-06 | 3,03E-02 |  | TG 58:4 | 8,50E-01 | 6,05E-02 |
| SM (d16:1/18:1) | 2,53E-02 | 3,03E-02 |  | TG 58:6 | 5,24E-01 | 7,06E-01 |
| SM (d40:1) | 8,90E-03 | 2,08E-02 |  | TG 58:9 | 1,34E-01 | 6,69E-01 |
| TG 42:0 | 1,15E-03 | 1,44E-04 |  |  |  |  |
